# Past, Present and Future of Bacillus Calmette-Guérin Vaccine Use in China

**DOI:** 10.3390/vaccines10071157

**Published:** 2022-07-20

**Authors:** Junli Li, Jinbiao Lu, Guozhi Wang, Aihua Zhao, Miao Xu

**Affiliations:** 1 Division of Tuberculosis Vaccine and Allergen Products, Institute of Biological Product Control, National Institutes for Food and Drug Control, Beijing 102629, China; nifdclijunli@163.com (J.L.); lujinbiao@nifdc.org.cn (J.L.); tbtestlab@nifdc.org.cn (G.W.); 2 Key Laboratory for Quality Research and Evaluation of Biological Products, National Medical Products Administration (NMPA), Beijing 102629, China; 3 Key Laboratory of Research on Quality and Standardization of Biotech Products, National Health Commission (NHC), Beijing 102629, China

**Keywords:** BCG vaccine, TB, MTB

## Abstract

The BCG vaccine is prepared from a weakened strain of *Mycobacterium bovis* (*M. bovis*), a bacterium closely related to *Mycobacterium tuberculosis* (MTB), which causes tuberculosis (TB). The vaccine was developed over 13 years, from 1908 to 1921, in the French Institut Pasteur by Léon Charles Albert Calmette and Jean-Marie Camille Guérin, who named the product Bacillus Calmette–Guérin (BCG). BCG, the only licensed vaccine currently available to prevent TB, is given to infants at high risk of TB shortly after birth to protect infants and young children from pulmonary, meningeal, and disseminated TB. The BCG vaccine, one of the safest and most widely used live attenuated vaccines in the world, recently celebrated its 100th anniversary (from 1921 to 2021); its record of use in preventing TB in China is also approaching 100 years. In 2022, a new century of BCG vaccine immunization will begin. In this article, we briefly review the history of BCG vaccine use in China, describe its current status, and offer a preliminary outlook on the future of the vaccine, to provide BCG researchers with a clearer understanding of its use in China.

## 1. Introduction

In 1900, Léon Charles Albert Calmette (1863–1933, French physician and biologist) and Jean-Marie Camille Guérin (1872–1961, French veterinarian, bacteriologist, and immunologist) at the Institut Pasteur in France conducted research on tuberculosis (TB) vaccines and found that it was difficult to produce a homogeneous suspension of *Mycobacterium tuberculosis* (MTB) when cultivated on glycerinated-potato medium. Subsequently, they tried adding ox bile to the medium to eliminate the tendency of MTB to clump, and they were surprised to find that this subculture resulted in a reduction in the virulence of the organism in guinea pigs and calves [1]. This accidental discovery led them to start investigating this attenuated tubercle bacillus for use in TB vaccines [2,3].

Starting in 1908, they isolated virulent *Mycobacterium bovis* (*M. bovis*) strains from a cow with tubercular mastitis in a glycerinated-bile potato medium and then subcultured them at roughly three-week intervals. By 1921, after some 230 subcultures were carried out over the preceding 13 years [4,5], *M. bovis* was successfully attenuated through the loss of nine crucial genes involved in pathogenicity or virulence, including Rv3871, PE35, PPE68, ESAT-6, CFP-10, Rv3876, Rv3877, Rv3878 and Rv3879c. The researchers found that this attenuated *M. bovis* failed to produce progressive TB when injected into guinea pigs, rabbits, cattle, and horses [6]. At Guérin’s suggestion, they named it Bacille Bilie Calmette-Guerin; later they omitted “Bilie” and thus BCG was born [2].

On 18 July 1921, Benjamin Weill-Halle (1875–1958) assisted by Raymond Turpin (1895–1988) administered a dose of BCG to a 3-day-old infant by the oral route (boosted on days 5 and 7 after birth) and found no adverse effects. Weill-Halle subsequently tried the subcutaneous and intradermal routes in other infants, but the local adverse reactions were opposed by the parents of the vaccinated infants. So, oral vaccination with BCG emulsion was commenced. By 1924, 664 infants were given oral BCG vaccination without serious complications, which greatly encouraged Calmette and Guérin [7]. Subsequently, the Institut Pasteur began mass production of the BCG vaccine for use in the medical community. Between 1924 and 1928, 114,000 infants had been vaccinated using the BCG vaccine [8]. In 1931, Calmette and Guérin set up a special laboratory to prepare and research BCG vaccines.

The introduction of the BCG vaccine marked an important advance in the history of TB control and brought optimism to the fight against TB, especially in endemic areas. Between 1924 and 1960, the Pasteur Institute delivered BCG cultures to more than 50 laboratories around the world. To date, BCG remains one of the most widely used vaccines worldwide and has been administered to over 4 billion individuals with astonishing safety records [9,10]. Other than BCG, no other vaccines are available to prevent TB, and of the many new candidates in the pipeline, none are close to market use. There are currently several mainstream BCG vaccines in use worldwide produced by different manufacturers. In addition to the China National Biotech Group (CNBG) in the Chinese market, the main manufacturers in the international market are Merck, Sanofi Pasteur, Serum Institute of India, Intervax, GreenSignal Bio Pharma Private Limited (GSBPL),and Japan BCG Laboratory (JBL). Each BCG vaccine is produced differently and is thought to have characteristic properties, such as the proportion of live cells per dose of vaccine which ranges from 50,000 to 3 million [11]. Although BCG was obtained by attenuated culture from a strain of *M. bovis*, due to its different passages and culture methods, strains in various countries in the world have formed many substrains with different phenotypes and genotypes [12,13]. Currently, over 90% of vaccines in use worldwide are prepared from the following strains: French BCG Pasteur 1173 P2 strain, BCG Danish 1331 strain, Russian BCG-I strain, Glaxo 1077 strain derived from the Danish strain, BCG Tokyo 172-1 strain, and Moreau RDJ strain [14]. Each strain has a different reactogenicity profile; the Pasteur 1173 P2 and Danish 1331 strains are known to induce more adverse reactions than the Glaxo 1077, Tokyo 172-1, or Moreau RDJ strains. In 2019, 88% of children worldwide received BCG vaccination during their first year of life [15]. To limit strain variation during vaccine production, the first World Health Organization (WHO) reference reagents for BCG vaccines of sub-strain Danish 1331, Tokyo 172-1, and Russian BCG-I were established in 2009 and 2010. These reference reagents cover the major proportion of BCG vaccine strains currently in production and supplied by the United Nations Children’s Fund (UNICEF) after prequalification for their global use. The National Institute for Biological Standards and Control (NIBSC) distributes the reagents as a WHO collaborative center [14,16]. 

As one of the countries with a high burden of TB, China introduced the BCG vaccine to prevent TB as early as 1928. BCG has thus been around for 100 years and has been used in China for almost as long. After a long period of development, the Chinese BCG vaccine also displays unique characteristics compared to other BCGs in the world. As the history of the BCG vaccine begins a new century, we summarize the history, current situation, and future development of the BCG vaccine in China, so that researchers can have a clearer understanding of this field.

## 2. Introduction of the BCG to China

In 1928, the Chinese Ministry of Health (the predecessor to the National Health Commission of the People’s Republic of China) convened the Health Construction Committee to adopt a proposal for the introduction and implementation of the BCG vaccine to prevent TB. In 1929, the BCG strain was sent to China and kept by Song Kouo-Ping (1893–1956, a bacteriologist from Yangzhou, Jiangsu province), then a professor of bacteriology at the School of Medicine in Shanghai Aurora University who had studied under Calmette. This was the first time that the BCG strain was introduced by the Chinese government. However, the world was shocked by the “Lübeck City BCG tragic disaster” in Germany in 1930, in which 72 of 251 infants died after receiving three oral doses of BCG vaccine contaminated with virulent MTB (Kiel strain), and another 173 developed pulmonary tuberculosis (PTB) but recovered after being infected [17,18,19]. As news of the “Lübeck disaster” spread around the world, Calmette and Guérin were the objects of considerable criticism and both came under great strain, as the safety of the BCG vaccine was questioned. In August 1930, at the Oslo meeting of the International Union against Tuberculosis, Calmette defended himself and received a great ovation. While the report of the German inquiry exonerated BCG as the cause of the disaster, confidence in the BCG vaccine had been undermined.

In 1933, Dr. Liang Wang (1891–1985, a physician from Chengdu, Sichuan Province), after studying with Calmette at the Pasteur Institute, brought back a French BCG strain to Chongqing, China, and prepared BCG vaccines for oral administration by following the culture manufacturing method of the Pasteur Institute. From 1934 to 1936, Dr. Wang vaccinated over 248 infants with the locally-produced BCG vaccine [20]. Therefore, Dr. Wang is considered to be the pioneer of childhood BCG vaccination in China. During this period, Dr. Fei-Fan Tang(1897–1958, a microbiologist from Liling, Hunan Province) of the National Epidemic Prevention Bureau (the predecessor of the National Vaccine and Serum Institute) and Dr. Yong-Chun Liu (1897–1953, a microbiologist from Yangzhou, Jangsu Province) of the Pasteur Institute of Shanghai also cultivated and produced BCG vaccines in 1936 and 1937, respectively [21,22]. Dr. Liu used his product to vaccinate local children. However, limited by the Chinese economic development at that time and various other reasons including World War II (1939–1945), in the 11 years since starting, he only managed to vaccinate 7500 children. Eventually, even though BCG administration was safe and had no obvious side effects, the BCG strain was lost, as it could not be preserved [23].

As the effectiveness of BCG in preventing TB was recognized, large-scale BCG vaccination began in Europe. From 1945 to 1948, over 8 million infants and children were vaccinated with BCG in Austria, Czechoslovakia, Finland, Greece, Hungary, Italy, Poland, and Yugoslavia [24]. To combat the TB epidemic, UNICEF and the WHO collaborated in 1947 to promote BCG vaccination worldwide. This massive BCG vaccination campaign was known as the “Joint Enterprise” [25]. In October 1947, the Ministry of Health of the Government of the Republic of China selected Zheng-Ren Chen (1914–1992, a microbiologist from Changsha, Hunan Province) from the National Epidemic Prevention Bureau, Xi-Hua Wei (1913–1982, a microbiologist from Ningde, Fujian Province) of the Epidemiological Prevention Laboratory of the Central Health Laboratory, and Zong-Yao Zhu (1913–1998, a physician from Tianjin) of the Tianjin Tuberculosis Prevention and Control Institute (the predecessor of the Tianjin Chest Hospital) as three trainees funded by the WHO Scholarship to study BCG production, standardization, and application at the Statens Serum Institut, Denmark [26,27,28]. The BCG seeds of the Statens Serum Institut were originally from the 423rd passage of the BCG strain provided by the Pasteur Institute in France in 1931, namely BCG Pasteur 423, which was mainly grown and serially passaged in bile potato medium or Sauton medium. From 1949 to 1966, the BCG strains were passaged exclusively in Sauton medium. In October 1948, Zheng-Ren Chen and Xi-Hua Wei returned to China and brought back the BCG Danish 823 strain, donated by the Statens Serum Institut, which was stored in the National Epidemic Prevention Bureau. This marks the second time that China officially introduced BCG [27,28].

## 3. BCG Strains in China

The specific strain is one of the important factors affecting the safety and efficacy of the BCG vaccine. Therefore, the choice of BCG strains has always been an important issue. It is difficult to determine which strain should be used on a global scale, although further detailed analysis of the genomics and immunogenicity of BCG sub-strains may provide an answer to this important question. The WHO and the International Union Against Tuberculosis and Lung Disease have been verifying which BCG substrains provide the best protection and recommend them for future vaccinations. China has also studied its own BCG strains, which ultimately ensures the uniqueness of BCG strain production based on preventing recovery of virulence while preserving an acceptable degree of “potency”.

### 3.1. BCG Strains in Modern Production

**Figure 1:**
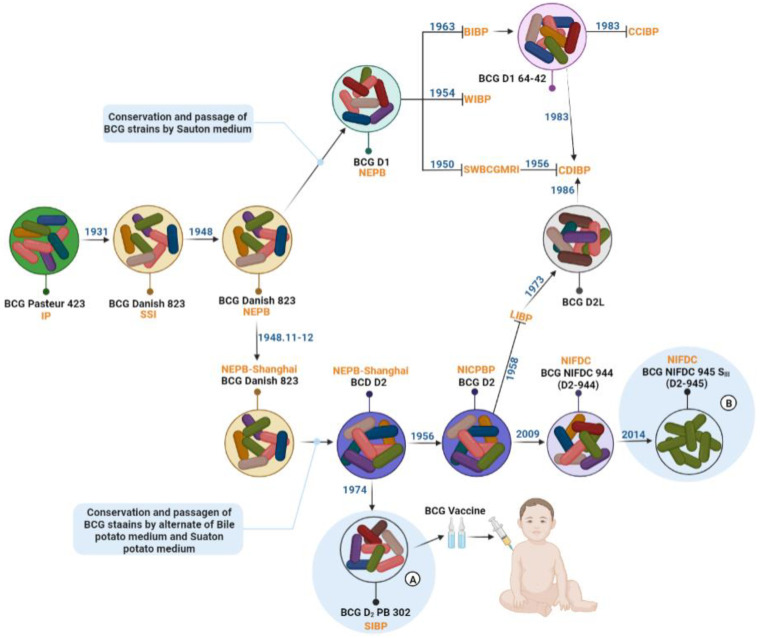
Chinese BCG genealogy and the movement of strains between labs. IP: Institut Pasteur, SSI: Statens Serum Institute, NEPB: National Epidemic Prevention Bureau, NEPB-Shanghai: National Epidemic Prevention Bureau-Shanghai, SWBCGMRI: Southwest BCG Manufacturing Research Institute, BIBP: Beijing Institute of Biological Products, WIBP: Wuhan Institute of Biological Products, CDIBP:Chendu Institute of Biological Products, CCIBP: Changchun Institute of Biological Products, LIBP: Lanzhou Institute of Biological Products, SIBP: Shanghai Institute of Biological Products, NICPBP: National Institute for the Control of Pharmaceutical and Biological Products, NIFDC: National Institute for the Control of Food and Drug. (**A**): BCG strains for modern production, (**B**): BCG single-cell clone strains.

In 1948, after the second BCG strain was introduced into China, a special BCG laboratory was established in the National Epidemic Prevention Bureau to start the development of the BCG vaccine [23,27]. In November 1948, Dr. Fei-Fan Tang returned to Shanghai from Beijing with the BCG strains. On 9 December 1948, Dr. Tang distributed the BCG strains to the National Epidemic Prevention Bureau-Shanghai (the predecessor of the Shanghai Institute of Biological Products) [27,29]. Since then, BCG researchers in Beijing and Shanghai have used modified Sauton medium for the serial passaging and preservation of strains. With sequential cultures and passages, the characteristics of the cultured progeny strains became significantly different from the BCG Danish 823 due to a change in the nitrogen source in the medium by the National Epidemic Prevention Bureau [29]. The strain with the new characteristics was called the Denmark–Beijing strain and designated BCG Denmark 1 (BCG D1). It was then distributed to various laboratories for strain preservation, research, and production until it was lyophilized in 1963 (D1 64-42) (Figure 1). 

At the same time, another strain was subcultured in Shanghai called the Denmark–Shanghai strain and designated BCG Danish 2 (BCG D2) (Figure 1). Researchers explored a variety of preservation and passaging methods for the BCG D2 strain. They found that, in the early stages, as BCG D2 was repeatedly transplanted in modified Sauton medium, the growth was slow and the biofilm that formed was rough. In the second stage, a Sauton potato medium was used for continuous passage to preserve the strains. After several generations, it was found that although the bacterial bryophytes grew rapidly, biofilm formation was slow and rough, which was unfavorable for further passage of the strain. In the third stage, glycerol potato medium was used for continuous passage, and it was found that neither the bacterial bryophytes nor the biofilm formation was ideal. In the fourth stage, BCG seedlings were alternately subcultured in bile potato medium and Sauton potato medium. The results showed that BCG seedlings grew well and formed thin, wrinkled, and elastic biofilms, which also met the requirement of subculture and amplification in Sauton medium. To ensure the stable preservation of various biological characteristics of the bacterial strain, the researchers freeze-dried this BCG strain and named it BCG D_2_ PB 302, where PB stands for Sauton potato medium and bile potato medium, and 302 stands for the generations of propagation of lyophilized BCG strain [30,31] (Figure 1).

In the 16 years from 1933 to 1949, the total number of children who received BCG vaccination in China was less than 100,000. This did not meet the TB prevention needs of a country with a large TB epidemic. After 1949, China established the National BCG Promotion Committee to vigorously promote BCG vaccination and established multiple BCG production institutions in Beijing, Shanghai, Lanzhou, Chengdu, Changchun, and Wuhan. In the two years from 1950 to 1952, BCG vaccination had been extended to more than 100 cities [32]. Approximately 600,000 children were vaccinated annually between 1950 and 1951. After that, the number of vaccinated people gradually increased, reaching about 4.75 million in 1958 [30]. In 1950, Dr. Liang Wang was charged with building the Southwest BCG Manufacturing Research Institute (which merged into the Chengdu Institute of Biological Products in 1956) and concurrently served as the deputy director of the institute and the director of the BCG vaccine department [23]. Since then, China has extensively carried out the selection and breeding of BCG strains.

In addition to the use of BCG D1 and BCG D2 strains since 1950, the strains used for BCG production in different institutions have also varied. In 1958, the Lanzhou Institute of Biological Products introduced the BCG D2 strain from the National Institute for the Control of Pharmaceutical and Biological Products [NICPBP, the predecessor of the National Institutes for Food and Drug Control (NIFDC)]. However, due to different passages, a special strain different from BCG D2, the Lanzhou strain, was eventually formed and designated D2L. In the 1950s, the Chengdu Institute of Biological Products used the original BCG D1 strain for production but found that BCG D1 was unstable during production. Therefore, they switched to the Lanzhou D2L strain for production after 1986. Another BCG vaccine production institution, the Changchun Institute of Biological Products, started to use BCG Brazil strains in the 1950s, and later changed to BCG D1 strains, and finally BCG Japan 172-1 strains in 1986 (Figure 1). Although the BCG strains used for production in laboratories in China all came from the same parent, BCG Danish 823, the passaged strains had mutated due to different culture and preservation methods, and the quality of the vaccines produced varied greatly. Some vaccines even produced lymph node abscesses and other phenomena after vaccination. This phenomenon led to confusion in the quality control (QC) and management of BCG vaccines.

To solve the chaotic approach to BCG production and management of different strains, in 1957, the Ministry of Health of the People’s Republic of China (the predecessor of the National Health Commission of the People’s Republic of China) officially issued an edict to carry out the selection and breeding of BCG strains nationwide. Since then, the NICPBP has cooperated with the BCG vaccine production institutions to conduct systematic and long-term comparative studies on different domestic BCG production strains, including biological characteristics, preclinical safety, immunogenicity, and effectiveness of animal protection. After 35 years of research, a strain with low inoculation side effects, BCG D_2_ PB 302, was finally determined to show good anti-TB protection effects in animals, good thermal stability as a freeze-dried vaccine, and good immunogenicity for large-scale population inoculation. It was established as the unified production strain of BCG in China, and vaccine production and a freeze-dried seed system were established for the production of BCG vaccines using this strain. In 1965, the WHO introduced the procedures for freeze-drying BCG vaccine and recommended the use of freeze-dried seed batch systems to preserve BCG strains to prevent variation [33]; the Shanghai Institute of Biological Products explored the establishment of freeze-dried seed batches to standardize the production of BCG in China. In 1974, the master seed lot and the working seed lot of BCG D_2_ PB 302 were freeze-dried and passaged simultaneously at home and abroad with a total of 1155 passages (D2-1155). Since then, China has established a complete system of freeze-dried seed batches for BCG vaccine production (Figure 1). In 1992, the Chinese Ministry of Health issued a “Notice on the Promotion and Use of BCG D_2_ PB 302 Strain”. Since 1993, China has switched to BCG D_2_ PB 302 strain as the only strain for BCG production, and it remains in use to this day [34]. 

In terms of genetic characteristics, BCG D_2_ PB 302 has deletions of RD1 and RD2, but not RD8, RD14, RD16, nRD18, or RD Denmark/Glaxo. In addition, BCG D2 PB 302 contains an IS6110 insertion sequence, and the SenX3-Regx3 locus has three tandem repeats. BCG D2 PB 302 strains are similar to Danish 1331 strains, however, RD Denmark/Glaxo is missing in Danish 1331, and its SenX3-Regx3 locus has only two tandem repeats. These genetic characteristics were stable within 12 generations from the working seed batch, and also proved that BCG D2 PB 302 is the descendant strain of Danish 823 [35].

### 3.2. BCG Single-Cell Clone Strains 

When BCG was first administered to humans to prevent TB in 1921, it was not possible to freeze-dry (lyophilize) BCG or preserve a stock of the strain in a freezer at −80 °C. The preservation of BCG strains was carried out by passing generations, but this traditional method of preservation was gradually abandoned after the freeze drying of different seed batches was recommended by the WHO. This means that the BCG vaccine has undergone different culture, passage, and preservation methods in different countries; this, over time, led to inevitable differences in immune effectiveness and attenuation levels among the BCG strains [36]. In addition, as early as in the 1970s, HG Dam also pointed out that although all the mother strains of BCG produced in countries around the world came from the Pasteur Institute in France, the BCG vaccine initially distributed was not a monoclonal colony, but a mixed colony [37]. It should be noted that the widely used 1973 lyophilized BCG Pasteur 1173P2 strain was also not derived from a monoclonal colony, which means that the BCG-producing strains used worldwide are still a complex of different BCG phenotypes [38].

To reduce the influence of passage on the effects of BCG vaccines, to understand the protection differences between the complex and the single-cell cloned BCG strains, and to standardize BCG vaccine production management, the BCG D2 strain was recovered by NIFDC in 2009 and passaged for the isolation of single-cell clones. It was passaged 944 times and was designated BCG NIFDC 944. In 2014, BCG researchers at NIFDC further isolated five single-cell clones from BCG NIFDC 944 using the microporous membrane method. Based on the culture and genetic characteristics of every single cell clonal strain, as well as the protective effect of MTB-infected animals, a single cell clonal BCG strain with good protective effects was selected and named BCG NIFDC 945 SⅢ. Following the rules of nomenclature for single-cell strains, NIFDC is the English abbreviation of the National Institute for Food and Drug Control, 945 means that the strain had been passaged for 945 generations, S means Single Colony, and the Roman numeral Ⅲ represents the third single cell clonal colony screened. Following good manufacturing practice (GMP) procedures, the single-cell cloned strain was continuously passaged and amplified three times in Lowenstein–Jenden medium, and the primary seed batch, main generation seed batch, and working seed batch were prepared and stored in the National Center for Medical Culture Collections (CMCC), with the strain preservation number CMCC94103. At present, BCG NIFDC 945 SⅢ is the only BCG vaccine production strain prepared by single-cell cloning and can be used for BCG prevention products, BCG derivative products, and recombinant BCG vector production strains.

## 4. BCG Production, Inspection and Vaccination in China

BCG bacterial culture mainly includes surface floating film culture and deep culture. Some European and American countries adopted the deep culture method, while most countries, including China, use the surface floating culture method, where the bacteria are cultured in static suspension on the surface of the liquid medium. The production process of the BCG vaccine in China can be divided into two stages: strain recovery and subculture, and biofilm collection and grinding. The working seed batches are continuously cultured and passaged on Sauton medium, and, after being amplified and passaged to a specific generation, the biofilm is collected by a biofilm collector, and the bacteria are ground and dispersed at low temperature. Then, an appropriate amount of non-allergen stabilizer is added to dilute to a certain concentration of the BCG bacterial stock solution, then quantitatively diluted to prepare the semi-finished products. The mixtures are divided into ampoules, freeze-dried, and sealed, which produces the BCG vaccine finished product [39,40,41]. The dose is 0.05 mg, and the number of viable bacteria should be no less than 1.0 × 10^6^ CFU/mg.

The first Chinese BCG vaccine developed by Dr. Liang Wang was an oral liquid formulation. China’s first national legal standard for biological products, the 1952 edition of the “Biological Products Regulations,” also listed the BCG vaccine as a liquid preparation. The 1959 edition, entitled “Biological Products Production and Inspection Regulations,” contained the provisional regulations for liquid BCG vaccines and freeze-dried BCG vaccines. The liquid BCG vaccine could be inoculated by intradermal injection, oral administration, and scratch inoculation. The 1979 edition of “Biological Products Regulations” added a freeze-dried BCG inspection scheme to the original protocols. The lyophilized BCG vaccine could be inoculated in two ways: dermal scratch and intradermal injection. The dosage of the dermal scratched BCG vaccine was 2–3 drops of 50–75 mg/mL. The dosage for intradermal injection was 0.1 mL of 0.5–1.0 mg/mL. The 1990 edition of the “Chinese Requirements for Biological Products” only included lyophilized BCG for intradermal injection. Since then, all three editions of the “Biological Products Regulations” or “Pharmacopoeia of the People’s Republic of China (Chinese Pharmacopoeia, Ch.P)” only included lyophilized BCG for intradermal injection. 

In 2013, technical specifications for vaccines for scheduled immunization were published in the WHO Technical Report Series (TRS) No. 979, in which the content on BCG QC included standards for seed batches, production processes, and finished products. The BCG quality standards in the “Chinese Pharmacopoeia III” (2020 edition) cover the WHO recommendations [42]. BCG strains, BCG bulk samples before lyophilization, and BCG freeze-dried vaccine ampoules representing each batch should be submitted to NIFDC for QC inspection following the requirements and standards, in addition to self-inspection by the vaccine manufacturer. QC inspection of BCG seed batches should include a bacteria identification test (analysis by culture characteristics and Multiplex PCR), bacterial purity test, bacterial virulence test, mycobacterium avirulence test, and immunogenicity test. QC inspection of freeze-dried BCG vaccine in ampoules should include a bacteria identification test (analysis by Ziehl–Neelsen staining and Multiplex PCR), physical characteristics test (appearance, volume difference, osmotic pressure, and moisture content), bacterial purity test, vaccine efficacy, viable bacteria content test, mycobacterium avirulence test, and thermal stability test. According to the inspection requirements for the BCG vaccine potency test listed in the “Chinese Pharmacopoeia,” the diameter of the local hardening reaction observed at the injection site in guinea pigs should be no less than 5 mm after 10 IU/0.2 mL TB- or BCG-PPD intradermal injection (the experimental guinea pigs must be tuberculin skin test [TST] negative, weigh 300–400 g, have the same sex, and inoculate 0.5 g of BCG 5 weeks before the group test). Since December 2001, China has required the supervision and management of the BCG vaccine and other scheduled immunization vaccines on a trial basis before each batch of products is reviewed, inspected, and certified by a designated drug inspection agency, to ensure the safety and effectiveness of BCG and other biological products. At present, two major BCG production institutions in China produce freeze-dried intradermal BCG vaccines for the scheduled immunization of children: the Shanghai Institute of Biological Products and the Chengdu Institute of Biological Products, CNBG, with a combined annual supply of no less than 20 million copies.

In 1974, the WHO created the Expanded Programme on Immunization to ensure universal access for mothers and children to routinely recommended vaccines; intradermal BCG vaccination at birth has been included in this program since its inception. However, national BCG vaccination policies vary broadly in terms of dosing and strains [43] and are cataloged in the BCG World Atlas [44]. The BCG vaccination policy in China has also progressed through different stages. In 1954, it was stipulated that “BCG vaccination is suitable for healthy infants within two months of birth and children over two months old and under 15 years old with a negative tuberculin skin test.” After 1957, the vaccination policy was amended by the addition of “except for the initial vaccination of newborns, re-vaccination every three years.” Before the 1980s, BCG vaccination in China was mainly concentrated in urban and rural areas with convenient transportation, and the strategy of regular centralized vaccination was adopted. At that time, the overall BCG vaccination rate was not very high. An epidemiological survey of tuberculosis in China in 1978 showed that in areas without BCG vaccination, the average tuberculosis infection rate of 7-year-old children was 7.3%. The prevalence of tuberculosis increased in children and adults. Therefore, a revised vaccination policy in 1979 stipulated that “intradermal vaccination is generally performed every four years for BCG revaccination, and dermal scratch vaccination is generally performed every three years.” In 1982, the BCG vaccination work plan further changed the revaccination age range to include the first grade of primary school until the first grade of junior high school. In 1986, the Ministry of Health promulgated a new basic immunization program for children; it stipulated that “BCG vaccination at birth, BCG revaccination at the age of 7, and a booster revaccination at the age of 12 for children in rural areas (urban children decide whether to strengthen the revaccination at the age of 12 according to the local tuberculosis epidemic situation).” In 1995, the WHO issued the “Statement on BCG revaccination for the prevention of tuberculosis” [45], which stated that the benefits of BCG revaccination could not be proven. After the statement was issued, countries that previously implemented BCG revaccination policies eventually stopped revaccinating. Similarly, the Chinese Ministry of Health notified the whole country in 1997 to stop BCG revaccination [46]. Currently, China’s vaccination policy is implemented per the “National Immunization Program and Guidelines on Childhood Immunization” (2021 edition). 

## 5. Prospects for Future BCG Use in China

The BCG vaccine has a significant, preventive effect on primary TB, miliary tuberculosis, and tuberculous meningitis in children, and the incidence rate of severe TB can be reduced by 92% after vaccination. Estimates of BCG vaccine protection against primary TB are influenced by the quality of research studies and whether the immunized population had been screened by a rigorous tuberculin skin test (TST) before vaccination; such protection is generally shown to be higher in neonates and by randomized controlled studies that utilize a rigorous TST test for vaccine efficacy. Based on the inference that MTB infection may block or mask the effect of BCG vaccination. Vaccinated populations previously infected with MTB or sensitized by non-tuberculous Mycobacteria (NTM) were associated with low efficacy of BCG against PTB and possibly miliary and meningeal TB [47,48,49,50,51]. It is worth noting, however, that individuals who completed latent TB infection (LTBI) treatment may have “unblocked” this “blockade” of the protective immune response induced by the BCG vaccine due to clearance of MTB compared with those who did not receive LTBI treatment [52]. The protection period of BCG vaccination is 10–15 years, and the effectiveness of BCG vaccination in preventing adolescent and adult TB has varied results [53,54,55]. The results of the fifth epidemiological sampling survey of tuberculosis in China in 2010 showed that the prevalence of active pulmonary tuberculosis gradually increased with age, showed an upward trend at the age of 20–25, and then reached a peak at the age of 75–80 [56]. The LTBI prevalence also showed a trend of increasing with age and the prevalence of LTBI in people aged 5 years old and above was 18.1%, and in people aged 15 years old and above was 20.3%, respectively. Moreover, a positive interferon-gamma release test (QuantiFERON [QFT]) was significantly associated with the absence of BCG scars in subjects under 20 years of age [57]. These all indicate that in addition to preventing tuberculosis during the BCG protection period, it is recommended that BCG vaccination can reduce the severity of the disease and reduce the infection rate of MTB. Similarly, the safety of the BCG vaccine in children and adults who are infected with HIV is unknown. Currently, the WHO recommends administering the BCG vaccine to asymptomatic, HIV-infected infants who live in high-risk areas for TB. BCG vaccines are not recommended for symptomatic, HIV-infected infants or for persons known to be or suspected of being HIV-infected if they are at minimal risk for infection with MTB [58].

The limited protective period conferred by the BCG vaccine may be related to the deletion of *M. bovis* genes encoding virulence proteins during the attenuation process, resulting in the weak immunogenicity of the BCG vaccine [59,60,61]. Therefore, novel types of TB-preventing vaccines should be developed to address the shortcomings of BCG, such as more immunogenic vaccines using recombinant BCG. Among them, BCGΔureC::hly (VPM1002), the only recombinant BCG vaccine that has entered Phase III clinical trials (ClinicalTrials.gov Identifier: NCT04351685), was genetically modified by the Max Planck Institute for Infection Biology to improve its immunogenicity by replacing the urease C encoding gene with the listeriolysin encoding gene from *Listeria monocytogenes* [62]. To overcome the short duration of BCG immunity, a novel vaccine is needed to strengthen the basic immune effect of BCG. As BCG has limited efficacy against untreated LTBI, a vaccine that can prevent the onset of LTBI should be developed, and the immune status of the population to be vaccinated should be taken into account.

Different BCG strains induce different immune responses in humans as well as animal models and the same strains perform differently in different locations [63,64]. Although, there are multiple hypotheses to explain the varying efficacy of BCG including genetic variability between different strains, loss of genes is essential for protective immunity, exposure to NTM, chronic helminth infections interfering with the immune response and variations in immunization methods [65]. However, no novel TB vaccine that offers greater protection than BCG has passed clinical trials. BCG remains one of the most cost-effective means for TB prevention in countries with a high TB burden, and likely will remain so for a long time. As a country with a high TB burden, China has a high rate of LTBI (a prospective cohort study of LTBI in rural China found that age- and sex-standardized rates of TST positivity [≥10 mm] ranged from 15% to 42%, and QFT positivity rates ranged from 13% to 20% [66].) and widespread vaccination of BCG (In the fourth national TB epidemiological sampling survey in 2000, BCG vaccination was analyzed in 59 survey sites. BCG scar screening was performed on 18,802 children aged 0–14 years. The number of children with a BCG scar was 12,799, and the incidence of a BCG scar was 68%, among which the incidence of a BCG scar was 94% in cities, 77% in towns, and 60% in rural areas. In addition, the number of TST-positive children with BCG scar was 2768, and the overall positive rate was 22%, among which the positive rate in cities, towns and rural areas was 28%, 31%, and 17%, respectively [67,68]). Therefore, to reduce the infection rate of MTB and the incidence of TB, a TB prevention system suitable for China’s national conditions should be established, where different immunization and prevention strategies are adopted for different populations with different infection and immune statuses. Until a novel TB vaccine to replace BCG is available, the BCG vaccine will continue to be administered to newborns. For adolescents and adults who are not infected with MTB or who lost their protection after previous BCG vaccination, the benefits of BCG vaccination may outweigh the risks if they are susceptible and at high risk.

Several further tasks remain for future BCG vaccine research. For the five-person per package BCG vaccine currently in use in China, attention should be paid to the genetic stability of the strain; the control of the production process should be bolstered; the differences between BCG vaccines produced domestically and abroad should be understood, and quality risk analyses should be carried out. At the same time, data monitoring and analysis should be conducted to ensure the stability of the product, quality, safety, and effectiveness of existing BCG products. Based on existing BCG production processes, the development of a single BCG vaccine per package manufacturing process is needed, to achieve minimum dose packaging unit production and enhance traceability. Clinical use of novel injection methods, such as needle-free or microneedle syringes, can improve children’s experience of inoculation. Reducing the risk of inoculation-related side effects and the incidence of abnormal reactions are also ongoing areas of research.

## 6. Conclusions

Although the efficacy of the BCG vaccine remains controversial (with the reported efficacy varying between 0% and 80%) [69], live attenuated BCG remains the only vaccine licensed for the prevention of TB in humans. The fundamental value of BCG vaccination is to prevent hematogenous spread once MTB infection is established. This is the basis for the recommendation to vaccinate newborns, currently followed in 141 of the 194 countries that use the BCG vaccine [70]. The incidence of pediatric tuberculous meningitis is very low in Western European countries, where vaccination of BCG is only a selective prevention strategy for vulnerable groups. However, the incidence of TB has increased over the past decade, from 3 million to 10 million new cases per year. This rise has been associated with an increase in HIV coinfection, especially in developing countries and countries with a high TB burden, such as India and China. The BCG vaccine is effective against the severe forms of TB and its use prevents a large number of deaths [71]. Thus, in highly endemic countries, the WHO continues to maintain mass BCG vaccination to prevent childhood tuberculosis, though it is acknowledged that this cannot break the “chain” of disease, which is sustained by adults with full-blown pulmonary forms. 

After 100 years of human use, BCG continues to be investigated against a variety of human diseases beyond its well-known roles in TB prevention. In recent decades, BCG has epigenetically and metabolically altered human immune cells to confer a heterologous immune response to immune challenges unrelated to mycobacteria. This is a known phenomenon in innate immune cells called “trained immunity.” A series of recent studies have even shown that the BCG vaccine may be useful in combating viral pandemics, such as COVID-19 and other diseases with high infection rates.

In summary, beyond its wide use as a vaccine and immunotherapy during the past century, BCG continues to serve as a valuable biological probe that has helped elucidate the fundamental properties of the human immune system.

## Data Availability

Not applicable.
